# Supplementary material for: Uncovering Novel Roles of miR-122 in the Pathophysiology of the Liver: Potential Interaction with NRF1 and E2F4 Signaling
Source: Cancers (Basel). 2023 Aug 16;15(16):4129. doi: 10.3390/cancers15164129 (PMC10453129; doi:10.3390/cancers15164129)
Supplement: Supplementary file 1 [file cancers-15-04129-s001.zip › Original WBs/2017-10-27 WB G6PD Huh7 miR122 OE Inhib.pptx]

## Slide 1
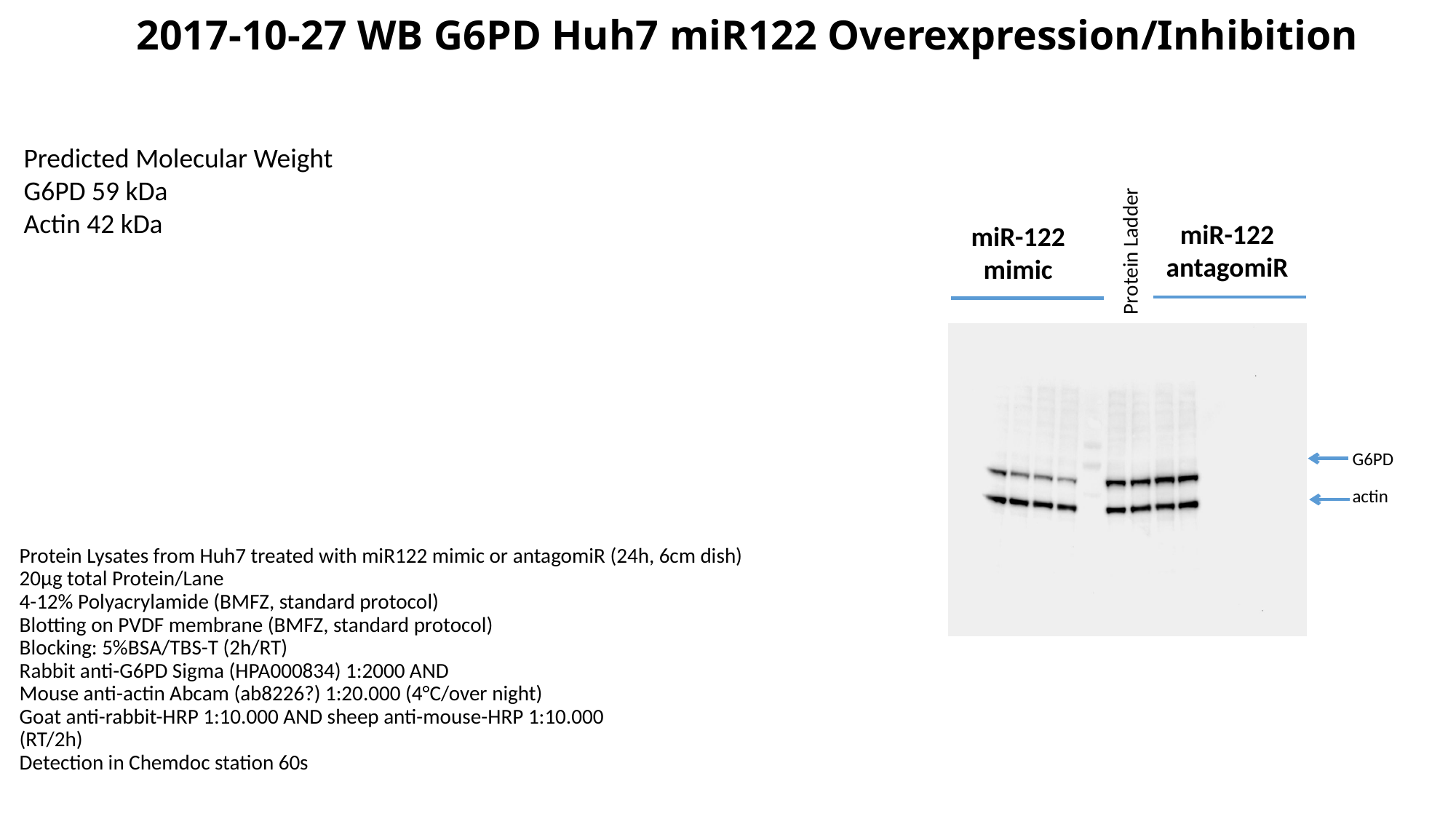

# 2017-10-27 WB G6PD Huh7 miR122 Overexpression/Inhibition
Predicted Molecular Weight
G6PD 59 kDa
Actin 42 kDa
miR-122 antagomiR
miR-122
mimic
Protein Ladder
G6PD
actin
Protein Lysates from Huh7 treated with miR122 mimic or antagomiR (24h, 6cm dish)
20µg total Protein/Lane
4-12% Polyacrylamide (BMFZ, standard protocol)
Blotting on PVDF membrane (BMFZ, standard protocol)
Blocking: 5%BSA/TBS-T (2h/RT)
Rabbit anti-G6PD Sigma (HPA000834) 1:2000 AND
Mouse anti-actin Abcam (ab8226?) 1:20.000 (4°C/over night)
Goat anti-rabbit-HRP 1:10.000 AND sheep anti-mouse-HRP 1:10.000
(RT/2h)
Detection in Chemdoc station 60s

## Slide 2
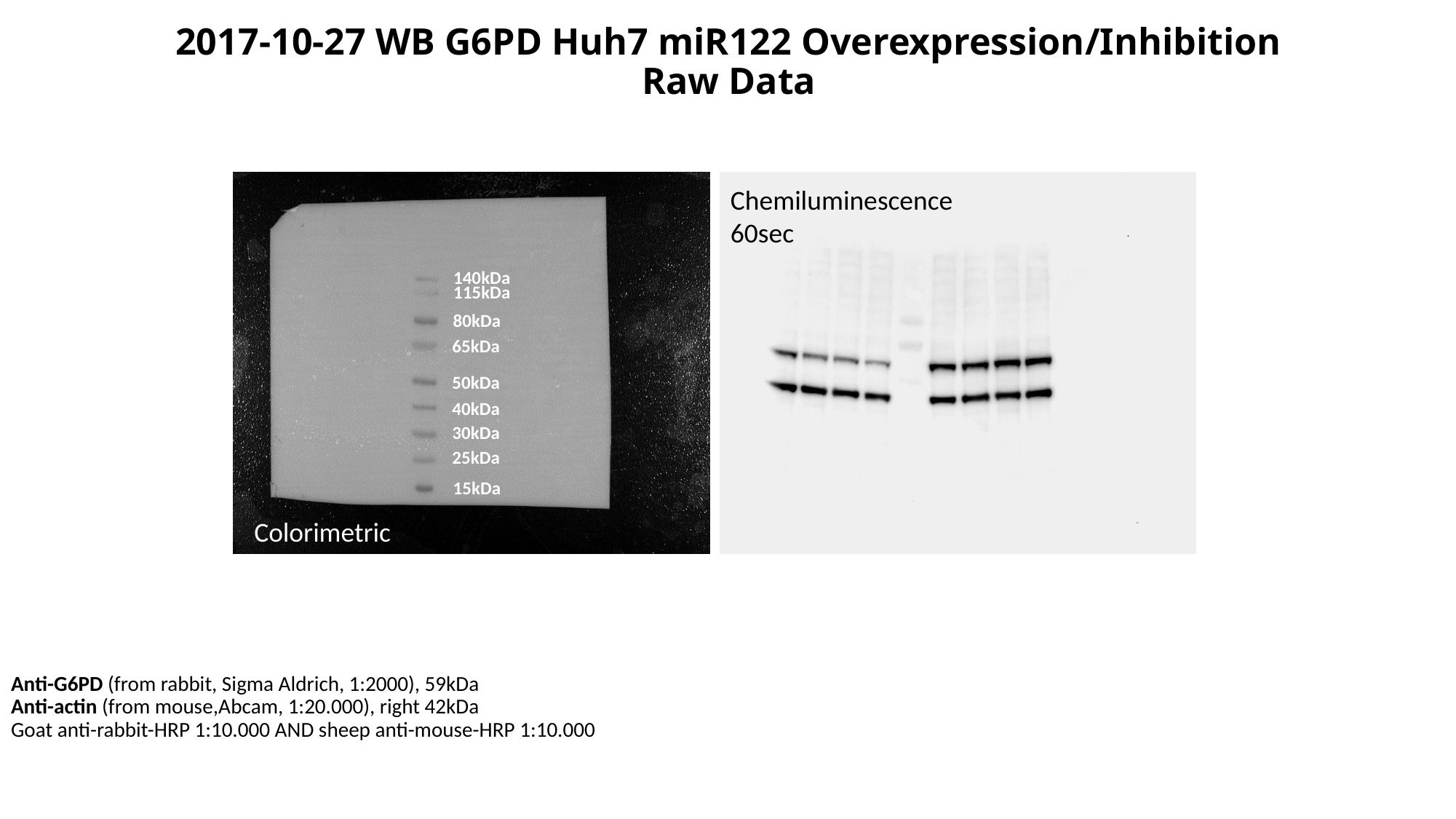

# 2017-10-27 WB G6PD Huh7 miR122 Overexpression/Inhibition Raw Data
Chemiluminescence
60sec
140kDa
115kDa
80kDa
65kDa
50kDa
40kDa
30kDa
25kDa
15kDa
Colorimetric
Anti-G6PD (from rabbit, Sigma Aldrich, 1:2000), 59kDa
Anti-actin (from mouse,Abcam, 1:20.000), right 42kDa
Goat anti-rabbit-HRP 1:10.000 AND sheep anti-mouse-HRP 1:10.000

## Slide 3
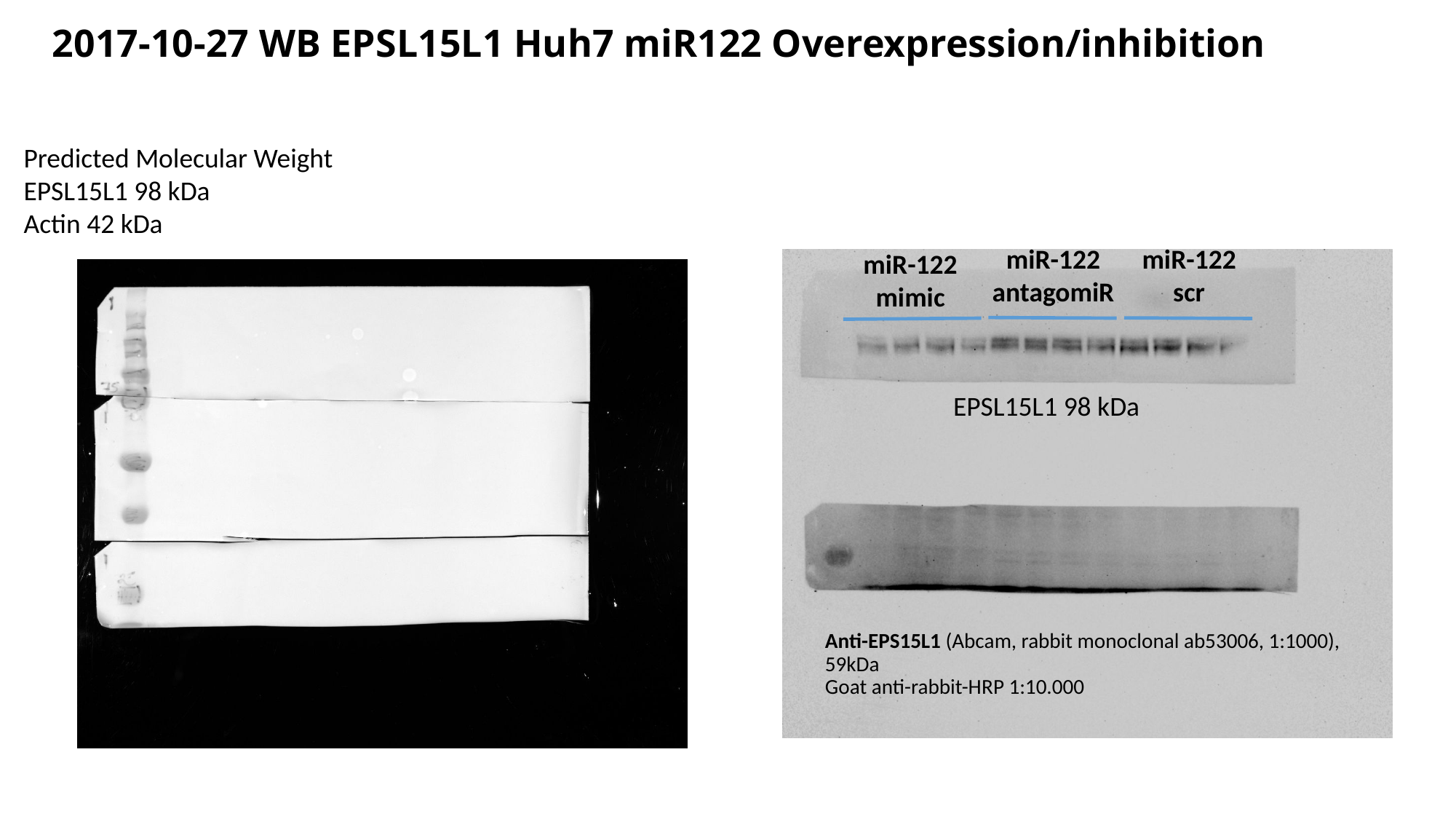

# 2017-10-27 WB EPSL15L1 Huh7 miR122 Overexpression/inhibition
Predicted Molecular Weight
EPSL15L1 98 kDa
Actin 42 kDa
miR-122 antagomiR
miR-122
scr
miR-122
mimic
EPSL15L1 98 kDa
Anti-EPS15L1 (Abcam, rabbit monoclonal ab53006, 1:1000), 59kDa
Goat anti-rabbit-HRP 1:10.000

## Slide 4
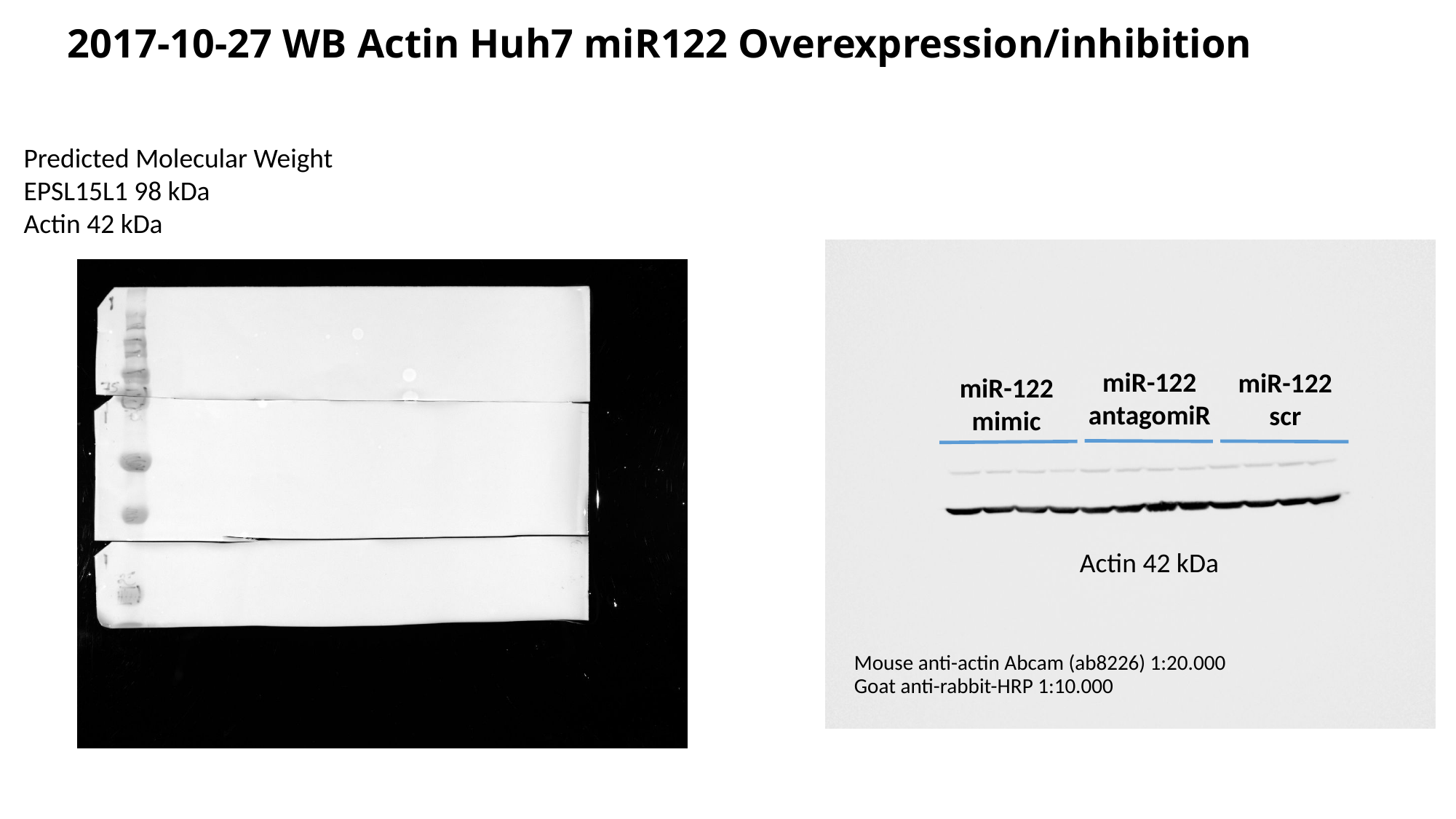

# 2017-10-27 WB Actin Huh7 miR122 Overexpression/inhibition
Predicted Molecular Weight
EPSL15L1 98 kDa
Actin 42 kDa
miR-122 antagomiR
miR-122
scr
miR-122
mimic
Actin 42 kDa
Mouse anti-actin Abcam (ab8226) 1:20.000
Goat anti-rabbit-HRP 1:10.000
