# Supplementary material for: Uncovering Novel Roles of miR-122 in the Pathophysiology of the Liver: Potential Interaction with NRF1 and E2F4 Signaling
Source: Cancers (Basel). 2023 Aug 16;15(16):4129. doi: 10.3390/cancers15164129 (PMC10453129; doi:10.3390/cancers15164129)
Supplement: Supplementary file 1 [file cancers-15-04129-s001.zip › Original WBs/WB Human HCC.pptx]

## Slide 1
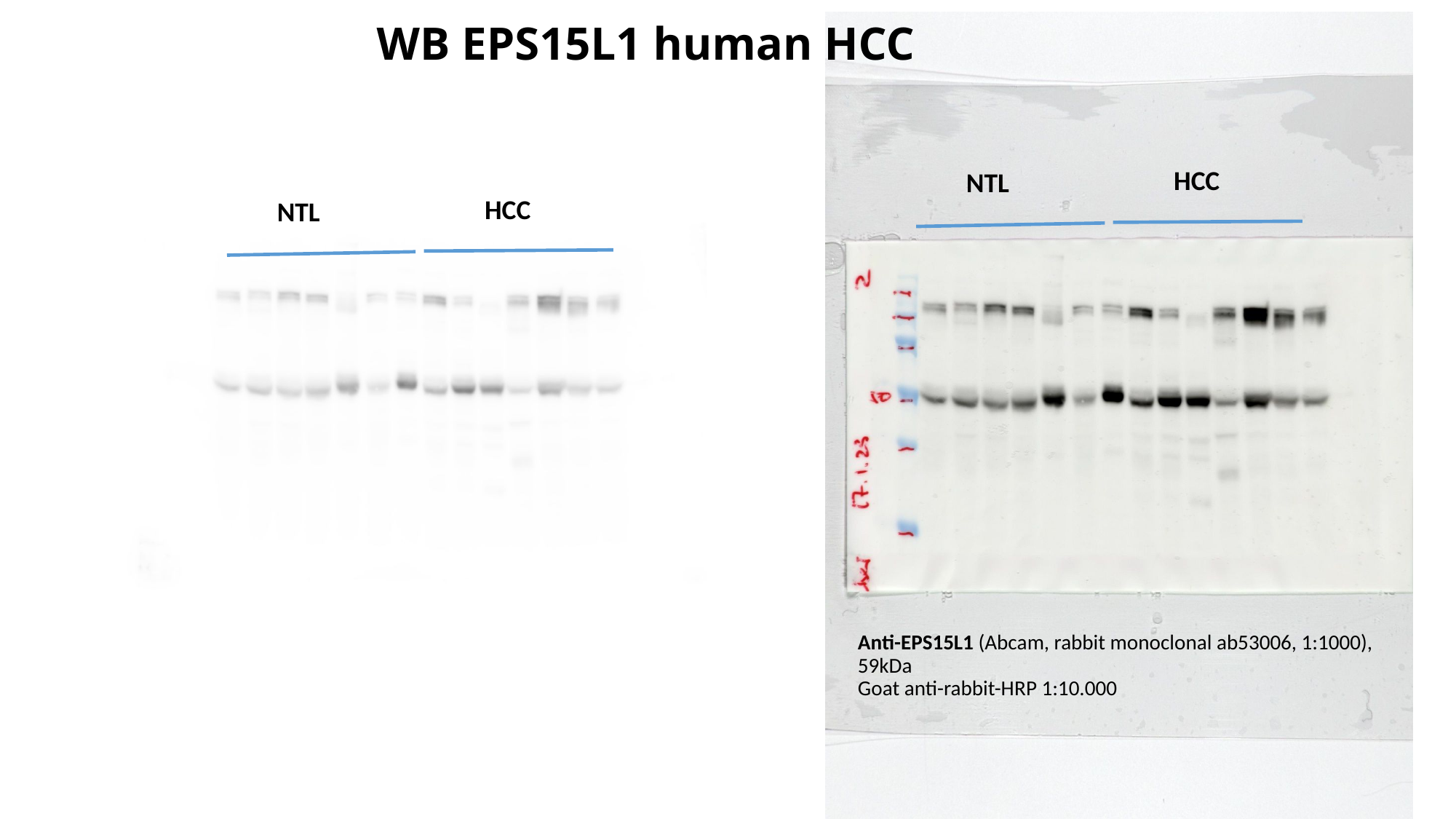

# WB EPS15L1 human HCC
HCC
NTL
HCC
NTL
Anti-EPS15L1 (Abcam, rabbit monoclonal ab53006, 1:1000), 59kDa
Goat anti-rabbit-HRP 1:10.000

## Slide 2
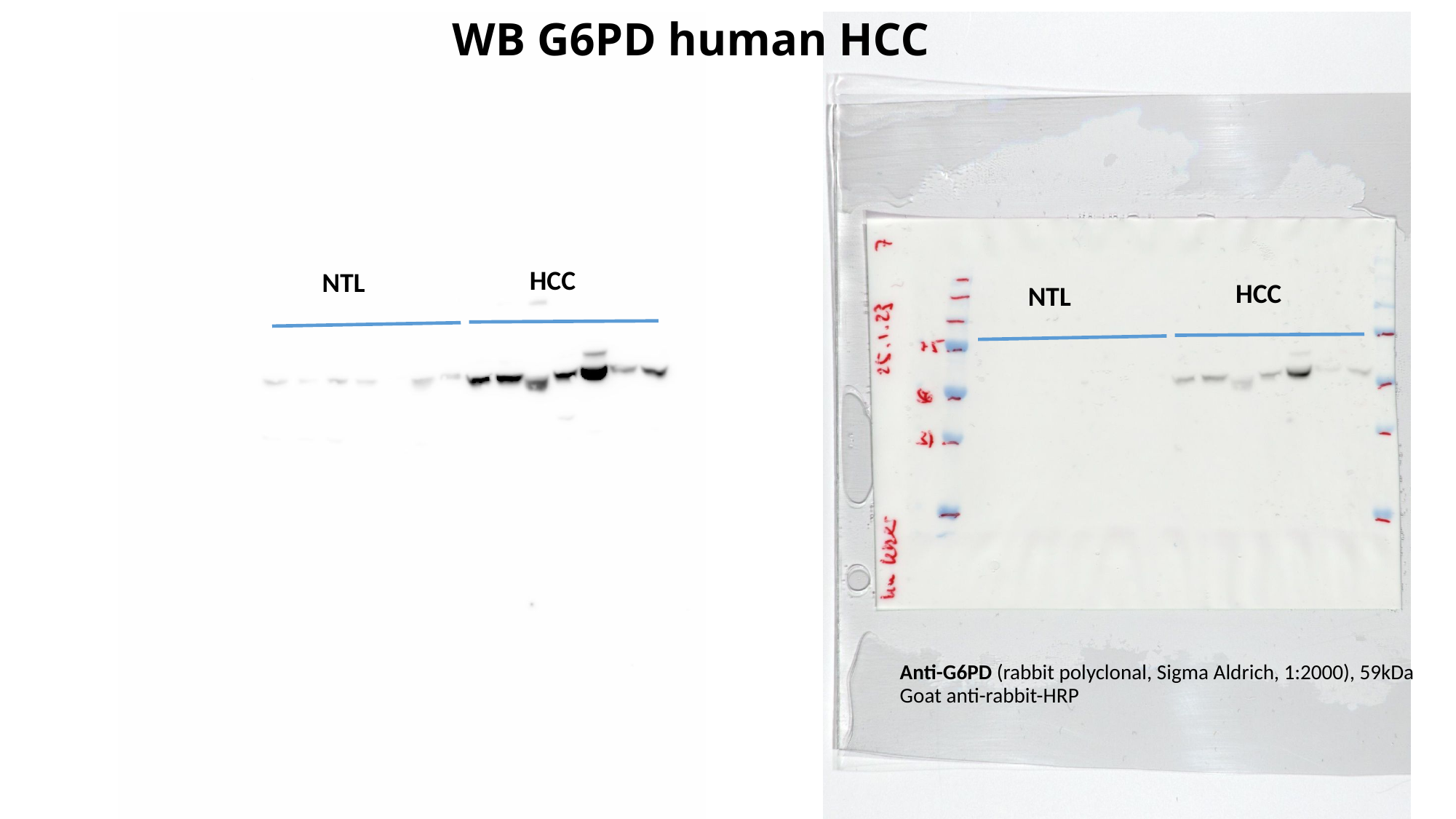

# WB G6PD human HCC
HCC
NTL
HCC
NTL
Anti-G6PD (rabbit polyclonal, Sigma Aldrich, 1:2000), 59kDa
Goat anti-rabbit-HRP

## Slide 3
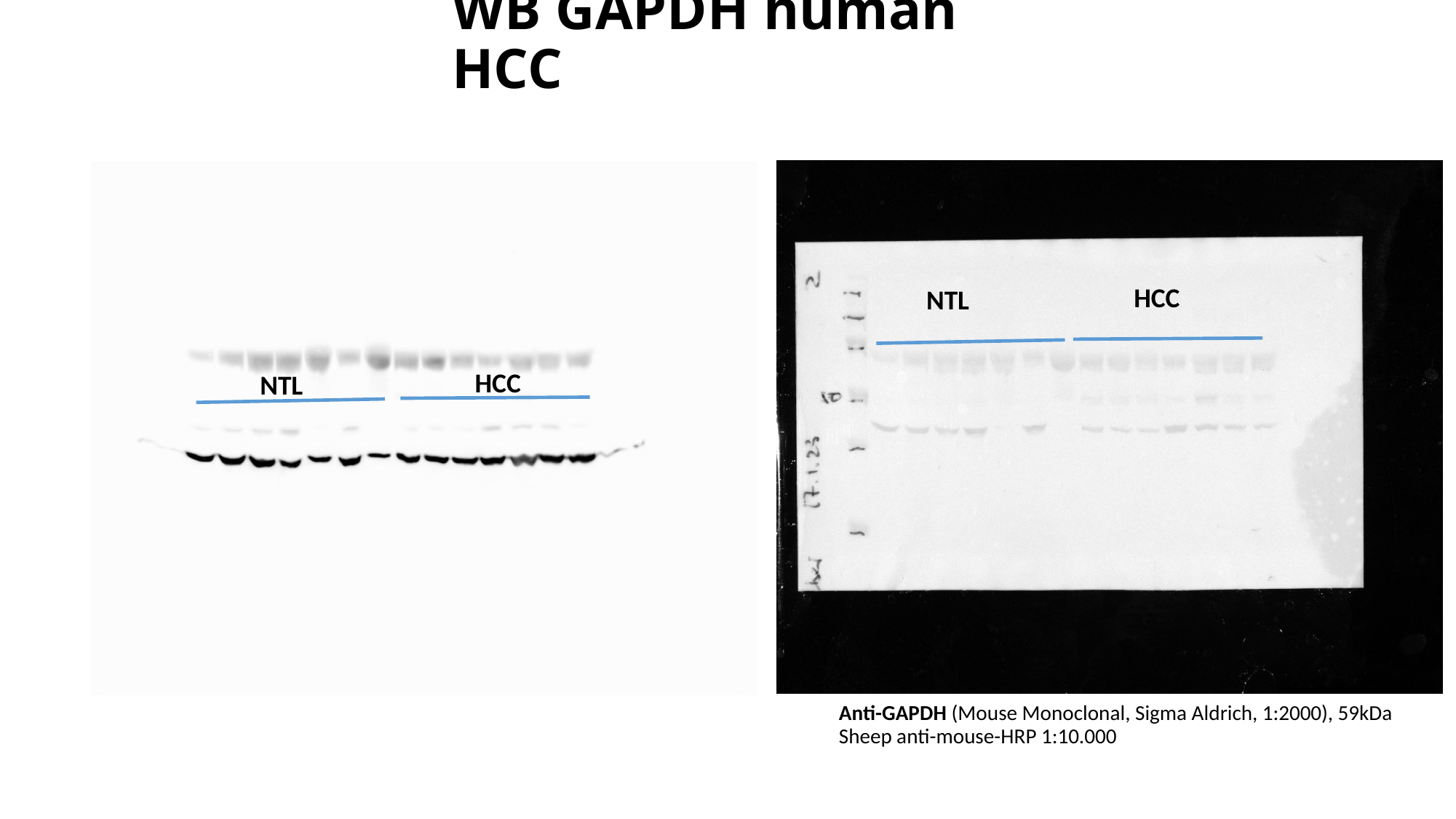

WB GAPDH human HCC
HCC
NTL
HCC
NTL
Anti-GAPDH (Mouse Monoclonal, Sigma Aldrich, 1:2000), 59kDa
Sheep anti-mouse-HRP 1:10.000
